# Molecular dynamics simulations of beta-2-glycoprotein 1 (β2GPI) reveal that post-translational modifications facilitate novel structures

**DOI:** 10.1038/s41598-026-52707-0

**Published:** 2026-05-20

**Authors:** Christophe J. Lalaurie, Paul A. Dalby, Thomas McDonnell

**Affiliations:** 1 Aging, Rheumatology and Regenerative Medicine, Division of Medicine, University College London, London, WC1E 6JF UK; 2 Biochemical Engineering, University College London, Bernard Katz Building, Gordon Street, London, WC1H 0AH UK; 3 Aging, Rheumatology and Regenerative Medicine, Division of Medicine, University College London, London, WC1E 6JF UK

**Keywords:** Autoimmunity, Protein flexibility, Antigenic, Biochemistry, Computational biology and bioinformatics, Structural biology

## Abstract

Beta-2-Glycoprotein I (β2GPI) is the main autoantigenic target of antiphospholipid syndrome (APS). β2GPI can adopt a number of structures; an extended J-shape, an O-shape and a transitional S-shape. It has also been shown previously that β2GPI can undergo a number of different post-translational modifications, altering its function and structure. It is unclear how the post translational modifications (PTMs) alter the progression through structures and if this alters the function of β2GPI. To study this we have chosen to use molecular dynamics simulations, providing fine atomistic details on the transition between structures, which can then be compared to findings published in the literature for validation. This study aimed to identify the potential effects of PTMs on the structure of β2GPI in-silico. Molecular simulations were carried out under standard physiological conditions (pH 7.4, 155 mM ionic strength, 1 bar pressure) for four models of β2GPI. These were the wild type, plasmin clipped (with terminal 8 amino acids removed), deglycosylated (with all four glycans removed) and reduced (with the terminal disulfide bond removed between C288-C326). Three repeats of 100 ns were carried out for each model. Plasmin cleavage and reduction generated structures absent from the wild type (WT). Plasmin cleavage generated a more compact structure with altered movement in Domain I (residues 1 to 60), whilst reduction produced unique elongated structures. These corroborated already published findings. Interestingly no simulation showed β2GPI circularise in these physiological simulations suggesting closing may not be an energetically favourable process. Molecular simulations of β2GPI identified structures only available to specific post translationally modified variants, and failed to show re-circularisation. Molecular simulations of β2GPI identified structures only available to specific post translationally modified variants, and failed to show re-circularisation.

**Supplementary Information:**

The online version contains supplementary material available at 10.1038/s41598-026-52707-0.

## Introduction

**Fig. 1:**
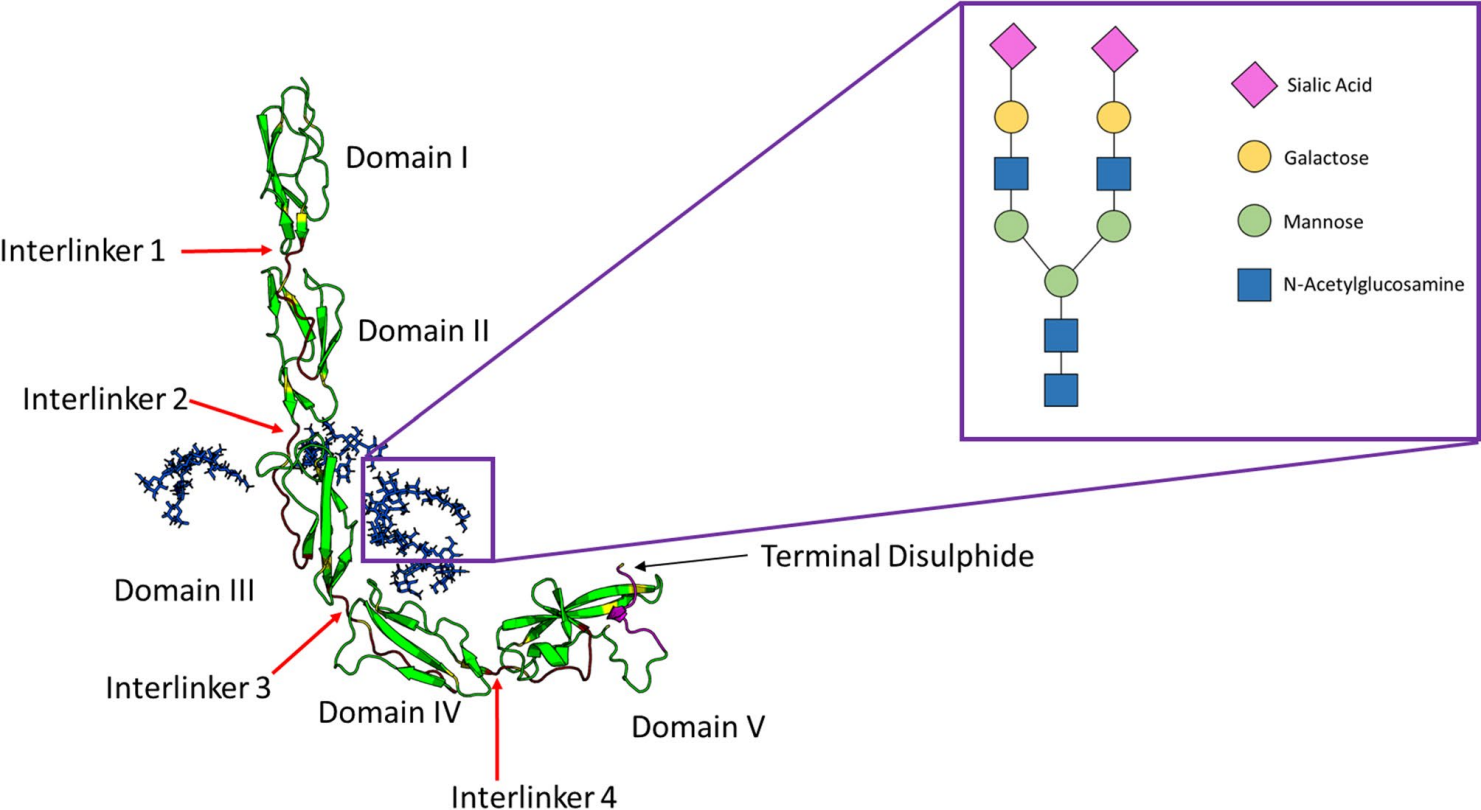
Linear model of β2GPI demonstrating the position of the interlinking regions (indicated by red arrows), the glycans (blue) including the glycan construction and branching (purple box). The disulphides are highlighted in yellow, including the reduced terminal disulphide (in the reduced model). The peptide liberated by plasmin cleavage is shown in magenta. This figure is a modification of the figure previously published in Lalaurie and Bradford et al. (2024).

Beta-2-Glycoprotein 1 (β2GPI) is a 50 kDa glycoprotein present in the serum at a concentration of 0.2 mg/mL. It consists of five domains arranged like beads on a string, four complement control protein (CCP) domains followed by a larger lysine rich domain (Fig. 1). There are four N-linked glycosylation sites commonly detected with a wide pattern of glycans^1^. There is also one potential O-linked site, although glycosylation at this position has yet to be found in humans. Several functions are proposed for β2GPI in the body^2^, and it has been shown to regulate both the complement and coagulation cascades, whilst other functions include lipopolysaccharides (LPS) scavenging and activating plasmin.

Structural studies of β2GPI have identified two dominant structures: a linear or extended J-shape and a circularized or compact form. The linear form has been characterised by both small-angle X-ray scattering (SAXS)^3^ and X-Ray crystallography^4,5^, while the circular O-shape has only been shown by scanning-electron microscopy (Scanning-EM)^6^. SAXS has also suggested the presence of an S-shape of β2GPI with a tilt between domains II and III (DII and DIII, residues 61–120 and 121–180)^3^, which may also explain the identification of a third form by ELISA using structurally specific monoclonal antibodies^7^. However, such a form has yet to be confirmed at atomic resolution. The mechanism and causes of transition between the observed circular and linear forms is not currently understood, although several mechanisms ranging from reduction^8^ to acetylation^9^ have been proposed to alter the structure of β2GPI.

The existence and importance of the circular (O) form of β2GPI remains an area of critical debate with conflicting evidence suggesting that it may be either a non-dominant transitional form, or may not exist at all in circulation^5^, although it has been identified in EM studies using plasma purified protein^6^. Kumar et al.^10^ spiked purified β2GPI into human plasma and identified no circular β2GPI by FRET or SAXS, suggesting that it does not circularise and thus calling into doubt the stability of the circular form in circulation. Crucially the protein was identified to be in an open form as evidenced by the binding of antibodies specific to a theoretically cryptic epitope in DI of β2GPI which drives the autoimmune disorder Antiphospholipid Syndrome (APS). However, it also remains possible that antibody binding drove the equilibrium towards the open form of β2GPI in APS through mass action.

APS is an autoimmune disorder characterised by thrombosis and pregnancy morbidity^11,12^. It is the leading cause of strokes in under 50-year-olds, and is the leading acquired cause of miscarriage. Although a rare disease (prevalence of 2.1 in 100,000 people)^13^, it has been the subject of significant study. While antibodies in APS can bind several domains, those targeting the N-terminal domain (DI) have been closely linked with pathogenicity and thrombosis in both humans and mouse models^14–16^. The underlying mechanism for APS patients developing auto-antibodies is poorly understood. The structure and conformers of β2GPI are likely to play a role in which antibodies are raised due to changes in epitope exposure.

The use of molecular dynamics to simulate and characterize protein structure dynamics at an atomistic level, over time, is now a well-established practice with modelling used in a number of such studies in structural biology^17,18^. This powerful tool has the capability to unlock the understanding of how proteins move, function and transition between conformers. The adoption of such technologies in rheumatology has, however, been slow with around 10 studies in the last 5 years.

Post-translational modifications of β2GPI can lead to a wide number of variants in circulation which are associated with differences in function. Interestingly, β2GPI can bind to plasminogen and aid in the activation of plasmin, which is then capable of cleaving β2GPI to generate plasmin-clipped β2GPI which has eight amino acids removed from the C-terminus. Although this has been recognized in vitro little is known about its function, with some studies suggesting that it loses the ability to bind to negative surfaces, alters endothelial activation^19^ and can inhibit angiogenesis^20–22^. Less is known about its structure, with evidence of structural change shown primarily by SDS-PAGE Reduced β2GPI, is produced by the reduction of a dihedrally-strained disulphide in the 5th domain which includes the terminal cysteine. Disulphides with similarly high dihedral strain energy are often associated with allosteric structural and functional change in a site distal from the disulphide. This has been studied extensively for both the structure and function of β2GPI, with an unusual level of backbone flexibility identified in previous studies^8,23,24^. Finally, differences that have been seen in the glycosylation pattern of APS patients in comparison to healthy controls with increased sialyation and different branching patterns^25^ being shown.

To begin to unravel the effect of these changes on the overall structure of B2GPI, we present a study using molecular dynamics of the above post-translationally modified β2GPI variants, starting from the structurally characterised J-shape, to interrogate the intra-protein associations which regulate the alteration of structure in β2GPI, and delineate the effects of structural changes on potential function.

## Methods

### Preparing molecular variants of β2GPI for molecular dynamics simulations (MDS)

A crystal structure model of β2GPI (PDBID: 1C1Z) was downloaded and prepared for molecular dynamics simulation using the Glycan Reader and Modeler^26–29^ at CHARMM-GUI website (http://www.charmm-gui.org/). Protonatable residues were edited on CHARMM-GUI for correct ionisation at pH 7.4. PDB 1C1Z represents the V247L variant of β2GPI and is one of the established crystal structures for β2GPI having been the backbone of previous structural work.

Modifications of β2GPI were carried out in silico using the CHARMM-GUI Glycan modeler platform. Plasmin cleavage was based on the high-resolution (2.86 Å) X-ray crystal structure of β2GPI (PDB ID: 1C1Z)^3^. To model the plasmin cleaved variant, amino acids 318–326 (the final 8 N terminal amino acids) were deleted, this was to ensure potential differences to merely the reduction of the terminal disulphide. The reduced variant was modelled by the removal of the disulphide bond between C326 and C288 and the deglycosylated model was created by the deletion of all glycans. All variants, barring the deglycosylated model, had four biantennary sialyated glycans (Man_3_GlcNAc_2_ core and two NeuNAc.Gal.GlcNAc antennae) attached to N143, N164, N174, N234 as detected by Kondo et al.^25^ and Baerenfaenger^30^, the wild-type β2GPI with these glycans is referred to as wild type (WT).

### Molecular dynamics simulations (MDS)

The TIP3P model was used for explicit water molecules. The cubic system size was designed to have at least 10 Å from the protein in each axis, and 0.15 M salt was added. The CHARMM36 force field was used^31,32^. All calculations were performed at 303.15 K. The particle mesh Ewald algorithm was applied to calculate electrostatic forces, and the Van-der-Waals interactions were smoothly switched off at 10 Å by a force-switching function^33^. A time step of 2 fs was used in all simulations. Initially, each system was shortly equilibrated in constant particle number, volume, and temperature (NVT) condition using CHARMM36^34^. To assure gradual equilibration of the system, positional restraints for backbone and side chain heavy atoms were applied and the restraint forces were gradually reduced during the equilibration. Each system was further simulated for 100 ns in 3 repeats using the CHARMM36 force field on the high-performance cluster, Kathleen, at University College London using NAMD2^35^. For the production NPT simulation, Langevin coupling coefficient was set to 1 ps^− 1^ and a Nosé-Hoover Langevin-piston^36,37^ was used to maintain constant pressure (1 bar) with a piston period of 50 fs and a piston decay of 25 fs. The time step was 2 fs and coordinates were saved every 100 ps. The electrostatic interactions were updated every 20 fs. The short-range nonbonded and electrostatic interactions were calculated with a cutoff of 12 Å. SHAKE was used to constrain all bonds involving hydrogen atoms. Convergence of the simulations for all systems was checked through the comparison of average root mean square deviation (RMSD) using VMD^38^. Each simulation was repeated three times and the data were averaged for analysis.

### Analysis

Analysis of RMSD, root mean square fluctuation (RMSF) and radius of gyration for MD trajectories was carried out using VMD (University of Illinois). Further analysis of dynamic cross correlation matrices (DCCMs) and principal component analysis (PCA) was carried out using R (Bio3d package).RMSD was analysed using VMD and the starting minimised structure for each variant was used as the reference for calculation. RMSD was used to determine movement from the starting structure, RMSF was used to determine movement from the average structure, the reference for which was determined by VMD. This combination allowed the detection of both progressive and repetitive movements whilst highlighting the areas with most fluctuation. PCAs were run using R to overlay trajectories and determine divergent structures, an elbow plot was used to determine the required number of clusters, values were chosen when the plot began to plateau. For composite PCAs containing all the trajectories from different variants, trajectories were trimmed of amino acids and glycans to allow the overlapping of structure, this was carried out after the trajectory and as such did not affect the movement of the molecule. Pathway analysis was carried out by monitoring how the clusters of structures changed over time and noting the frequency with which certain structures progressed into other clusters, this was carried out using Microsoft Excel. Salt bridges were analysed using a combination of VMD and Excel to monitor their creation over time. Finally, radius of gyration (Rg) and DCCM analysis were carried out in RStudio to monitor both the overall shape/diameter of the protein (Rg) and to detect correlations between distant amino acids within the protein (DCCM). Graphs were prepared in Prism Graphpad (v10).

## Results

### Generation of MD construct

Plasmin cleavage was simulated by the deletion of the final eight C-terminal amino acids, whilst reduction was simulated through removal of the C-terminal disulphide bond (C326-S-S-C288), and deglycosylation was generated through the deletion of all glycans. The starting point for the MD can be seen in Fig. 1 (1C1Z). Glycans (blue) were supplemented using CHARMM-GUI to match the previously detected patterns of 4-Di branched glycans as shown in Kondo et al.^25^. The Biantennary pattern was selected as it was the most dominant when analysed by Baerenfaenger et al. in 2019^30^, this pattern also reduced potential positional isomers of branching glycan variants.

### PCA analysis reveals structures unique to specific variants

A first pairwise RMSD analysis (Supp. Figure 2) was used to confirm a stable state had been reached for each simulation. To achieve this, every frame within each model was used as a reference structure to measure RMSD from in every other frame of that model. This revealed that all simulations reached a stable state by the end of the 100 ns simulation time, as evidenced by a patch of low RMSD values within the top right corner of each repeat versus itself. This analysis also confirmed that the two most sable models were the wild type and the plasmin clipped, with the highest RMSD values, both within and between repeats, were reached in the deglycosylated and reduced simulations. This confirms the importance of the glycans and of the disulfide bonds for the total stability of this molecule.

**Fig. 2:**
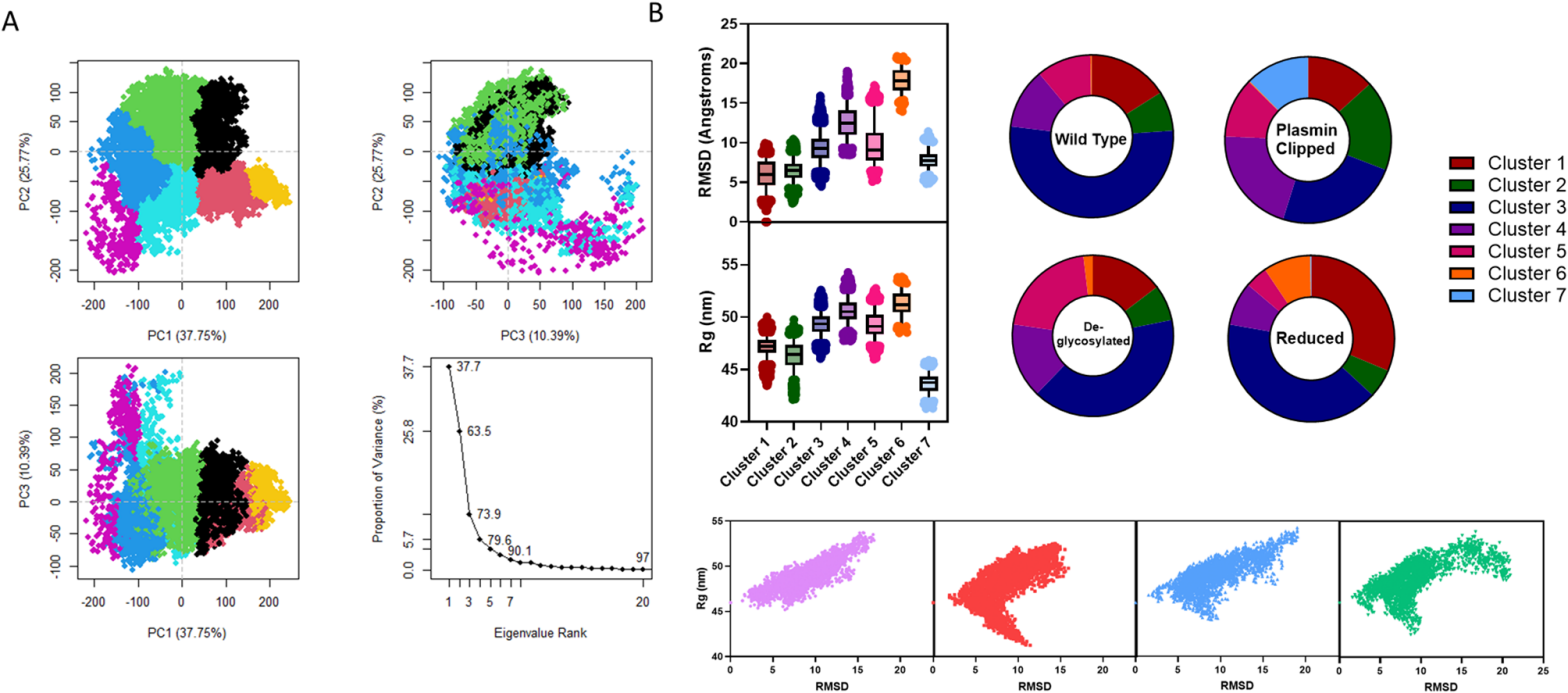
PCA analysis was undertaken to identify clustering. (**A**) To analyse for unique structures within the simulations, all simulations were clipped to 317 amino acids long (to match Plasmin Clipped) and merged. Optimal clustering was seen with a K of 7, which isolated structures unique to certain models (clusters 6 and 7). (**B**) Clusters 6 and 7 have very different RMSD and Rg profiles, with cluster 6 representing the highest change and most elongated whilst cluster 7 shows a lower RMSD but a smaller Rg. These clusters are dominated by Reduced and Plasmin Clipped, respectively, with structures which are largely specific to those two variants. Similarly, cluster 1 is dominated by the reduced form whilst cluster 5 is dominated by the deglycosylated form. Cluster 4 is also heavily present in the plasmin clipped form in comparison to other forms. Further granularity can be seen when plotting RMSD vs. Rg ( L to R Wildtype, Plamsin Clipped, Reduced, Deglycosylated), where a significant correlation (*P* < 0.001) between RMSD and Rg are seen in all cases, whilst the relative R values are different with WT showing 0.81, Deglycosylated 0.793, reduced 0.63 and the lowest with plasmin clipped at 0.5791.

To compare structures between variants, MD trajectory files were clipped to the common 317 amino acids and the coordinates of all frames combined across variants were subjected to PCA (Fig. 2). An elbow plot showed that seven clusters of structures captured the majority of the unique structural conformations explored, while a Scree plot showed that using the first four principal components for the clustering captured 80% of the variance. As such K was set to 7 and the first four principal components were used for the separation of the frames into those seven clusters (Fig. 2A). Interestingly, some structures were only explored by particular variants, with cluster 7 appearing primarily in Plasmin Clipped β2GPI (> 95% of cluster 7 frames are from the plasmin clipped trajectory) whilst cluster 6 appeared primarily in reduced β2GPI (80% of frames in cluster 6 were from the reduced trajectory) (Fig. 2B). Interestingly, certain clusters were more prominent in different variants, with plasmin clipped β2GPI dominating clusters 2, 4 and 7. In contrast, reduced β2GPI was predominantly observed in clusters 1 and 3, whilst deglycosylated β2GPI was predominantly found in clusters 3 and 5. The wild type β2GPI simulations populated over 50% into cluster 3, with an even split of the remaining frames across clusters 1, 2, 4 and 5. By contrast, plasmin clipped β2GPI had the lowest population in cluster 3, suggesting an early move away from the natural conformations populated by the wild type β2GPI simulation. This shift away from the balanced wild type distribution is mirrored in all the modified forms, suggesting that post-translational modifications may lead to changes in the relative populations of structural forms even where the modification does not lead to any new structural conformations not already observed in the wild type β2GPI.

**Fig. 3:**
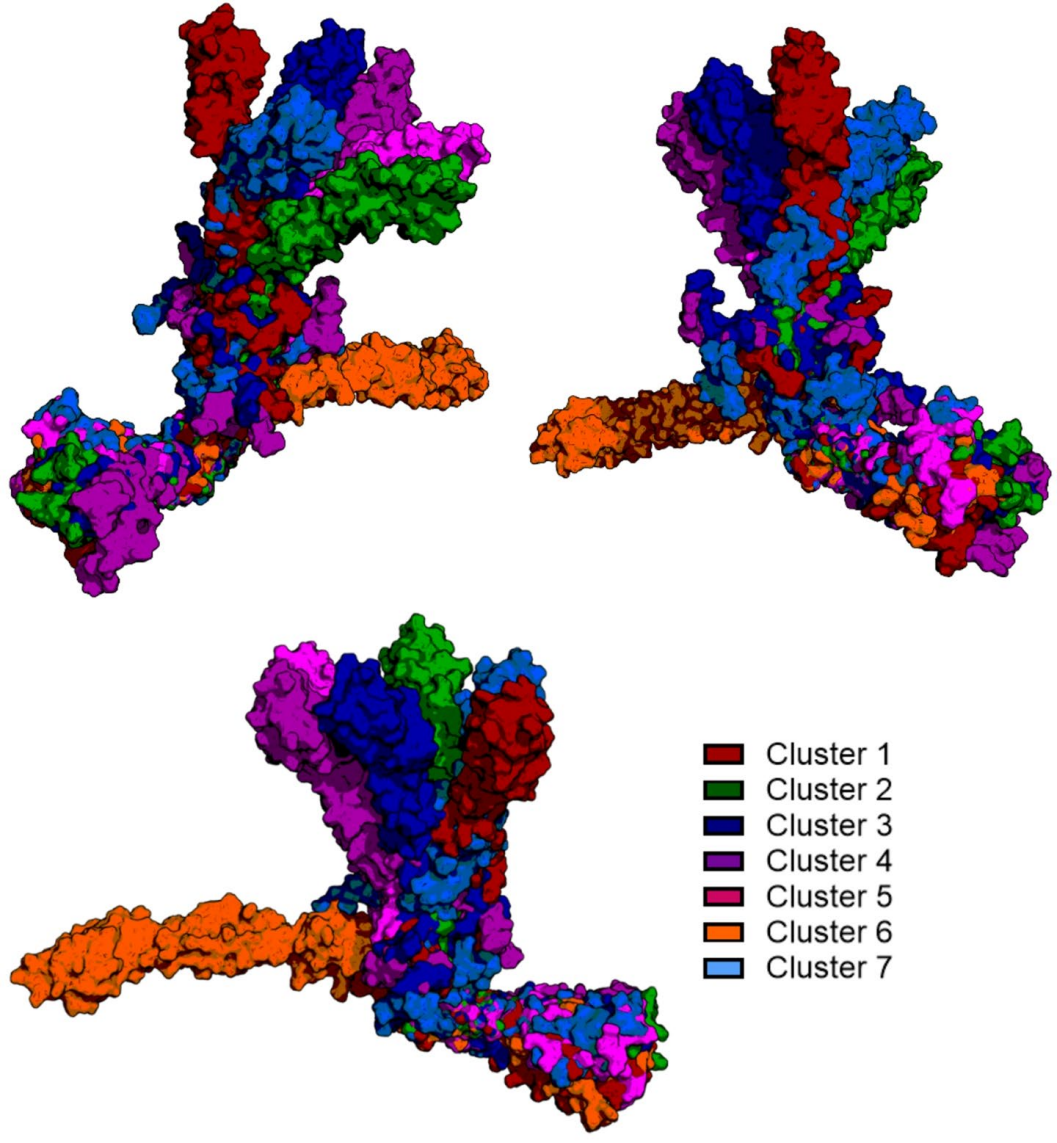
Overlays of the structures highlight the extreme movements in clusters, the powder blue and green are dominated by plasmin clipped and show a unique curve to the right, while the orange is the reduced and represents structures in cluster 6, and is orientated flat away from the reader in the image on the left. These were aligned by DIII to highlight the movement both above to DI and below to DV. Interestingly, these also show torsional movement with the faces of the green and blue structures differently exposing the DI epitope compared to those of clusters 1 and 2. This suggests the movement may be more subtle than simple RMSD measurements can capture.

Representative structures from the major clusters are shown in Fig. 3, and aligned via DIII alone to maximize visualization of the conformational changes between them. Two of the structures show notable movement of the relative position of DI, for example, with similar positioning for clusters 2 and 7 (tipped to the right in Fig. 3 in powder blue and green). These two clusters were mostly observed in simulations of the plasmin clipped β2GPI suggesting this movement is induced by alterations in the 5th domain. The most extreme change in conformation was seen for cluster 6 where the protein became almost linear (orange), and was represented mostly by the reduced β2GPI. This also accounts for the most extreme variation in RMSD and Rg seen for cluster 6 in Fig. 2B. The pink (cluster 5) and purple (cluster 4) also appear to be relatively similar in their movements for DI-DII, and are most prominent in the deglycosylated form accounting for > 30% of that simulation whilst it is just 22% of wild type. Further cluster analysis highlighted the divergent nature of cluster 7, which occupied the lowest Rg values and also low RMSD values (Fig. 2B), which is explained by the compact form seen in Fig. 3 (powder blue). Similarly, cluster 2 which was the other plasmin clipped dominated cluster shows a low Rg and low RMSD suggesting the movement in plasmin clipped is consistently towards a compact form. Cluster 6 which contains the most extreme forms found in the reduced construct shows the higher RMSD and highest Rg as suspected, though some outliers from cluster 4 show similar Rg but far lower RMSD suggesting different forms of opening of the structure are taking place. No circular form was seen in any simulation suggesting the protein will not re-circularise in these conditions at least within the 100 ns timescale.

### PCA and DCCM analysis highlights correlative nodes

**Fig. 4:**
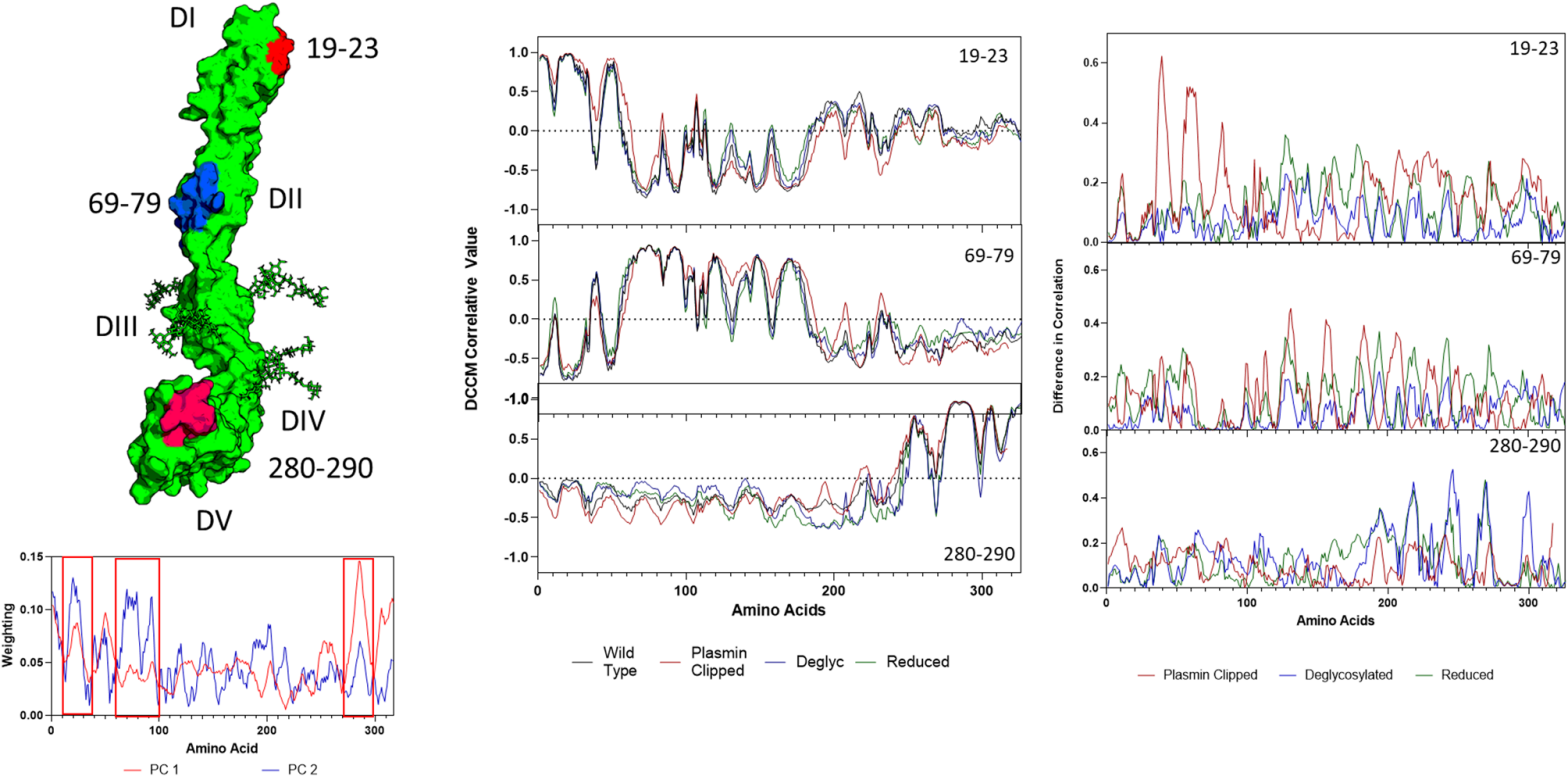
Plotting PCA loadings for PC1 and PC2 showed here regions of interest (19–23, 69–79 and 280–290) driving clustering. Plotting these on the structure shows an interesting alignment spread across the protein, when investigating the correlation by DCCM, interestingly 19–23 shows a loss of negative correlation in D1 for the plasmin clipped, but a gain of negative correlation in the regions of 110–150 and 230–240 suggesting the differences from the cleavage are widely seen. In the region of 69 to 79 differences are seen for all modified forms with the plasmin clipped gaining the most positive correlation with 120–160 and then increases in correlation in the region of 190–210. All modifications became dysregulated in the regions of 260 to the terminus. The most obvious differences are seen in 208–290 where plasmin clipped becomes extreme compared to all variants with changes in the region of 190–210 where correlation is lost, and a gain of negative correlation across the 1st 2 domains. Similarly reduced and deglycosylated both become much more positive regulating in DII and DIII whilst generating novel negative associations in the regions of 180–250. This highlights the 190–230 region as crucial to the structural change seen, the sequence immediately following Interlinker 3. This was better demonstrated when the absolute difference from the wild type was plotted which highlighted regions of extreme change.

To further identify amino acids driving structural change in β2GPI we re-examined the PCA and plotted the amino acids which drove the clustering (Fig. 4). Principal component 1 was driven mostly by the region of F280-Y290 whilst principal component 2 was influenced by regions in DI and the DI-DII interlinking region. We also looked at the dynamic cross-correlation matrices (DCCMs) of each model to assess the impact of these PTMs on the internal dynamics of the protein (Fig. 4). Interestingly the K19-E23 region saw the most correlative change relative to WT in the 1st domain specifically in the plasmin clipped molecule. Both the reduced and plasmin clipped models show large change in correlation relative to WT in the G69-T79 region. The F280-Y290 region shows the most change relative to WT for the deglycosylated and reduced proteins. Mapping these regions onto the protein (Supp. Figure 3) highlighted the global changes in correlation for the plasmin clipped protein, whilst the alterations in the deglycosylated are much more spatially restricted. These sites were plotted across the protein with their relative distance to correlation region (Supp. Figure 3) and plasmin clipped β2GPI showed correlative changes across the DI-DII interlinking regions with increased distal correlations too in the DIV-DV regions associated with the K19-E23 site whilst the G69-T79 site altered the DIII-DIV linker – something much less prominent in other modified forms. Both the other modified forms showed greater correlations in the DIV-DV region. Interestingly when averaging the correlation between domains, a similar pattern is seen with the greatest alterations seen in DI for plasmin clipped β2GPI whilst deglycosylated β2GPI shows the greatest difference in DIV-V (Fig. 4 and supp. Figures 4 & 5). Finally intra-domain DCCM was calculated to identify local changes in correlation, (Fig. 4 and Supp. Figures 4 & 5) with the greatest differences seen in the increased regulation of DV internally for plasmin clipped β2GPI when compared to all other variants. This was not mirrored in reduced β2GPI, suggesting this was not an effect of the loss of the terminal disulphide alone. Alignment of the structures suggested these movements may be due more to overall conformational change, i.e. a twisting across an axis, with increased twisting in the plasmin clipped form rather than specific changes driven by these regions (Fig. 3).

**Fig. 5:**
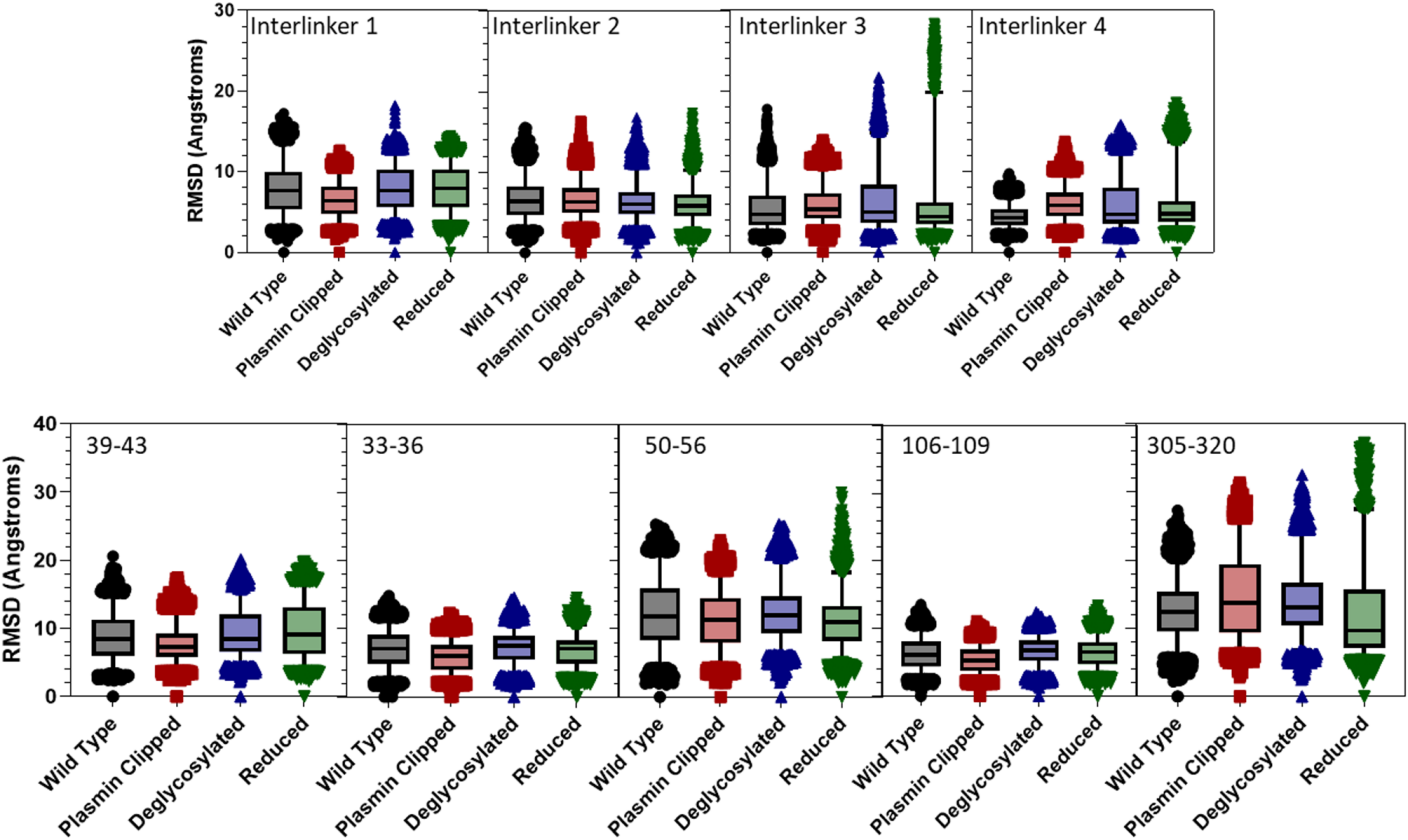
Investigation of flexible regions within β2GPI showed a markedly reduced flexibility in linker between DI and DII in the plasmin clipped protein, whilst no other model was visibly different. All groups contain > 3000 frames (min. 3008, max. 3073). In contrast, interlinker 2, which connects DII to DIII showed decreased flexibility in both the glycosylated and the reduced forms. Interlinkers 3 and 4 (connecting DIII to IV and IV to V respectively) showed increased movement in all the modified forms, with the most extreme profiles seen in the reduced form, however, the most consistent increase were seen in the plasmin clipped and deglycosylated forms. The highest mean average movement in both the 3rd and 4th interlinker regions was seen in the plasmin clipped form. This suggests any structural movement seen in the molecule likely comes from alterations in these hinge regions. We also explored the RMSD in the antibody binding regions (lower panel) and the surface binding regions of DV (K305-A320). The main epitope was more stable in the plasmin clipped species (39–43) whilst the same was seen for 33–36 which is believed to stabilise the region. Both deglycosylated and reduced protein showed more movement in the 39–43 and 33–36 regions than wild type. Again the 106–109 was more stable with lower RMSD in the plasmin clipped while higher movement was seen in the other modified forms. Plasmin clipped also had a lower RMSD than wild type for the 50–56 region which is potentially stabilising for the DI-DII interlinker which may explain the low RMSD in the upper panel. The plasmin clipped showed by far the highest consistent movement for the K305-A320 region which allows cellular binding, this would be in keeping with other data suggesting this species loses effective cellular binding potentially due to this increased movement. Similarly, reduced forms show a large distribution of values for this region, though the mean value is lower than the wild type control.

### Interlinking regions show marked change

Next, we investigated the inter-linking regions of the protein (Fig. 1) to assess how far these regions could explain the overall changes in structure. Interestingly the modified forms showed different profiles of movement in these interlinking regions (Fig. 5). In the DI-DII interlinker (interlinker 1), lower movement was seen in the plasmin clipped protein in comparison to all other constructs, this suggests maybe this structure is more stable throughout the simulation. In contrast the DII-DIII interlinker (Interlinker 2) shows generally lower movement in the reduced and deglycosylated structures than the non-modified (wild type) and plasmin clipped suggesting these two domains become less flexible in these constructs. The majority of movement is seen in the interlinker 3 and 4 regions. Interlinker 3 which sits between the DIII and DIV shows the greatest flexibility in the deglycosylated variant, however both this and plasmin clipped are noticeably increased compared to wild-type. This is also true of the region between DIV and DV (interlinker 4) with the highest average value seen in plasmin clipped. This highlights that different movement is associated with different structures and arises from different sites within the protein. Crucially it also highlights that interlinker 3 and 4 are allowed more freedom after the modifications allowing structural changes in relative domain positions rather than changes to the internal structure of the domains themselves.

### Sites of interest show modified movement

Investigation of sites of interest near the DI epitope and the DV lysine binding region revealed little global change, however some local minor changes were identified for the variants. For example, lower movement (RMSD) was seen for the plasmin clipped β2GPI compared to the wild type for the proposed epitope region (39–43) (Fig. 5. lower panel & supp. Figure 6). In previous research we found that the 106–109 loop appeared to stabilize the epitope and together presents the DI-II interlinker epitope^39^. Again, this loop showed lower movement for the plasmin clipped β2GPI while the other modified forms showed increased movement. This suggests that the modifications can lead to alterations of the epitope – which matches what has been shown in literature with increased antibody binding to plasmin clipped β2GPI^42^ and altered binding to reduced β2GPI. Similarly, there was a very large increase in the movement of the K305-A320 region which dominates binding to cellular surfaces. This also corroborates previous literature findings of decreased binding to cellular surfaces^42^. Interestingly this was not matched by the reduced-β2GPI which showed reduced mobility suggesting the changes are not due simply to the loss of the disulphide but destabilisation on the release of the peptide.

### Modifications drive alterations in salt bridge formation

Given the difference in movement in the sites of interest and the formation of novel structures, we also investigated salt bridge formation as a surrogate for stability. Interestingly reduced β2GPI formed more salt bridges across the whole protein (Supp. Figure 7) however, when focusing on the five most populous sites across the non-modified form, this increase was less obvious suggesting a more global formation for reduced β2GPI. The most common sites for salt bridge formation were in DI, DIII and DIV. Salt bridges formed in DI (E26-K19, E27-K44) were very different such that all modified forms showed fewer salt bridges in E26-K19, whereas only the plasmin-clipped β2GPI formed fewer salt bridges than the non-modified form at E27-K44. This suggests that the stability seen in the DI-DII interlinker region and the increased binding in literature is unlikely due to increased salt bridge formation in the 1st domain. Furthermore, deglycosylated β2GPI shows an increase in salt bridges at the E27-K44 site suggesting increased stability in this region. The other striking difference is in the D293-K276 salt bridge within DV which shows a dramatic reduction in the plasmin-clipped β2GPI which is likely due to the increased movement seen in the 5th domain in the clipped construct.

## Conclusions

Beta-2-Glycoprotein I is a complex serum protein capable of a wide array of different functions in the body from altering complement and coagulation to LPS scavenging and angiogenesis. It is also capable of adopting a number of conformations, most commonly an open J-shape and a closed O-shape^6^ although the existence of the circular form has recently been called into question. Relatively little is known about how β2GPI transitions between these structures in the body, or indeed the role of the independent structures within different systems. As such we conducted a molecular simulation with common post translational modifications, including glycosylation, cysteine bond reduction and plasmin cleavage including the removal of the cleaved peptide, using the validated high resolution crystal structure (PDB ID: 1C1Z)^3^ of β2GPI to investigate the effects that these modifications would have on protein structure. These modifications, specifically plasmin cleavage, are suspected of being capable of greatly changing the conformational space that β2GPI can access.

Our study represents the first in-depth molecular simulation of full length β2GPI edited with this range of post translational modifications, building on previous work from our group utilising plasmin cleaved β2GPI in MD simulations. The modifications selected included two common modifications (reduction and cleavage) and one theoretical modification (deglycosylation). The choice to remove the peptide generated by plasmin cleavage was taken to maximise the potential effects on the protein. We aimed through this to identify if the different functions of B2GPI when post-translationally modified, may be in some way governed by structural shift. The presence of the peptide post-cleavage is somewhat debated in the field with some documented cases of the peptide remaining^40^ whilst in other scenarios it has been shown to be cleaved^41^.

Plasmin clipped β2GPI shows a different structural profile with a smaller Rg, different movement in the interlinking regions and a more stable profile in the 1st domain. This is then corroborated by changes in the salt bridge formation in the 1st domain whilst PCA analysis identified structures unique to plasmin clipped β2GPI. Analysis of the epitope regions identified in literature also showed a large increase to the stability for plasmin clipped in these regions but a loss of stability in the cellular binding region in DV. These corroborate many findings in literature including a loss of cellular binding for plasmin clipped β2GPI^42^, and different structures seen through both IMMS and SAXS^43^. Interestingly it also implies altered antibody binding for this form of β2GPI as the sites selected in our analysis have all been shown through mutation studies to be associated with antibody binding. We recently showed increased antibody binding for the plasmin clipped form in our own dataset^43^. Similarly there are unique functions of plasmin clipped β2GPI, for example, β2GPI binds to Factor XI and inhibits activation by thrombin and factor XIIa, however, this is lost upon clipping^19^. Further to this, clipped β2GPI has been shown to bind to angiostatin 4.5, a kringle-heavy protein which is anti-angiogenic. Shi et al. also showed that the addition of β2GPI led to decreased endothelial cell proliferation, invasion and tube formation, an effect not seen for non-clipped β2GPI^19^. Our molecular dynamics data may explain these differences in function with cleavage shown to heavily change the structure of β2GPI leading to new exposed regions and different stability. These conformations may interact with receptors that non-clipped β2GPI cannot access and thus lead to altered signalling.

Reduced β2GPI has been discussed recently in several manuscripts^8,23^ and has been shown to have increased flexibility and potentially incorporate a different structure. Reduced β2GPI showed less movement in the linear form of β2GPI with patches of DI and DV showing more movement, however, it did form a unique structure which was detectable by PCA whilst also showing the most salt bridges in one cluster before the rapid loss of salt bridges across the simulation, mirroring previous data suggesting increased flexibility^8^. Interestingly the greatest differences seen in the DCCM for this variant were relatively local in comparison to the long-range alterations with the plasmin clipped. The majority of alterations also focused across the DIV-DV interlinking region potentially explaining the increased movement seen in previous studies^8^. Interestingly, reduced β2GPI has been linked with several different functions for example, reduced β2GPI can bind to platelets^44^ whilst the oxidised form inhibits this process, our data would suggest this may be due to stabilisation of the K305-A320 region which has been shown previously to be involved in the binding to platelets and has been highlighted in previous simulations of DV^45^. In our simulations this was notably more stable than wild type despite having a much larger distribution of values. Reduced β2GPI is protective for endothelial cells in the presence of hydrogen peroxide and oxidative stress. This suggests a wider function for reduced β2GPI as a vascular protector in the presence of free radicals. The immunogenicity of the reduced form is somewhat controversial with several studies finding different outcomes. Buchholz et al.^8^ showed increased specific anti-Domain I binding while Kumar et al. (2021)^23^ failed to show any increased binding, however this may be due to the use of an artificial monoclonal (MBB2) where Buchholz used purified anti-DI antibodies from multiple APS patients meaning the Buchholz work utilizes a polyclonal response. Similarly, they utilize two very different assay approaches, therefore differences are not unexpected. Our data supports the theory that no loss of antigenicity is seen, agreeing with the work of Kumar et al., however, it is hard to predict how the movement in the DI and increased stability may lead to increased binding, whilst alterations in association with regions proximal to the epitope are seen, again it is hard to predict the potential for these fluctuations on antibody binding meaning we cannot refute the findings of Buchholz et al.

Deglycosylated β2GPI is arguably the least clinically relevant variation as it has not been shown to exist in vivo, however, alterations in glycan patterns have been seen in APS patients regarding sialyiation, thus a thorough characterization of the effect of glycan variations beginning with complete deglycosylation has significant value^1^. Interestingly it showed a much larger Rg throughout which may suggest the glycans play a role in packing and stabilizing the J-shape and a loss of glycan alters the overall shape. Similarly, it showed increased alterations in the associations within DV, suggesting the loss of glycans may alter the movement of the 5th domain.

Whilst the work presented is theoretical, it validates many findings from previous work, from epitope exposure and movement through to physical structure determination. It is also important to note the smallest structures formed were seen in the plasmin clipped model and whilst the wild type did show some smaller structures it did not circularize. The simulations’ RMSD plateaued suggesting that the maximal space was sampled (Supp. Figure 8),. In our previous study^39^ we have shown that the O shape spontaneously opens, this simulation shows no evidence of re-circularisation before reaching a steady state, which corroborates the work of Ruben et al. and Kumar et al.^5^ who showed spiking B2GPI into plasma failed to trigger recircularization. This lends credence to the growing evidence that the circular B2GPI form may be unstable and may not be the dominant form in serum rather it may be either an expressed/inactive form or a form generated during transition. Similarly we made the choice to simulate using 1C1Z as, although it contains the V247L variation, it is a commonly circulating variant and is the predominant circulating variant in Asia^46^. Whilst more modern structures do exist (6VO6) at a higher resolution, this model was undergoing version changes as of the time of this study and did not undergo its SAXS PDB validation until after the conclusion of this work. It also contains the V247 residue which is dominant in Europe and as such represents a different model of β2GPI. Further work comparing, these mutations and their differences if of great interest, however, it did not fall within the scope of this study. Finally, we recognise the use of 10 Angstroms padding in the simulation box is a minimum and in some cases may lead to artefacts due to interactions with the periodic image. Given the range of movement seen in our simulations we know this has not happened in this case. Future work should include a larger box as a precaution nonetheless.

In conclusion, our molecular simulations have identified novel conformations which may help explain the different functions of these variants in vivo and inform future structural studies of β2GPI. While we have observed that the PTMs introduced to these models lead to a modified conformational sampling and unique structures for individual models, future work should include finer details such as the impact of these modifications on the charge distribution, the intra- and inter-domain interactions between residues, and attempt to explain how these changes can bring about the observed modification of the proteins’ dynamics.

## Electronic supplementary material

Below is the link to the electronic supplementary material.

Supplementary Material 1

## Data Availability

All data is available at reasonable request post discussion with the corresponding author.
