# Supplementary material for: Molecular dynamics simulations of beta-2-glycoprotein 1 (β2GPI) reveal that post-translational modifications facilitate novel structures
Source: Sci Rep. 2026 May 20;16:22987. doi: 10.1038/s41598-026-52707-0 (PMC13392268; doi:10.1038/s41598-026-52707-0)
Supplement: Supplementary file 1 — Supplementary Material 1 [file 41598_2026_52707_MOESM1_ESM.docx]

**Supplementary Figures**


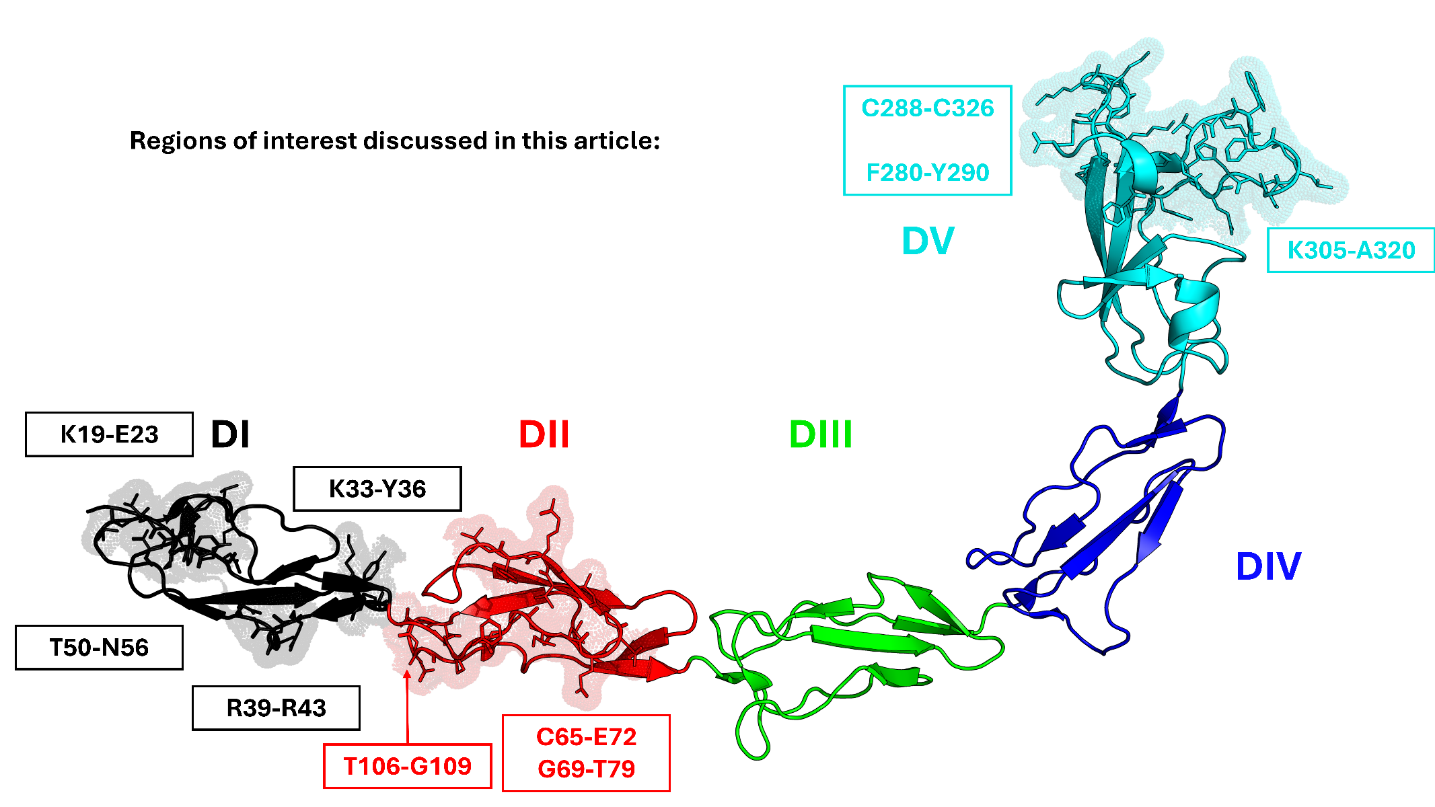


Supp. Figure 1: Annotated cartoon representation, minus the glycans for clarity, of the regions of interest discussed in this article. Within DI we have included the region of high correlation K19-E23, the loop linked to general regional stability K33-Y36, the established epitope R39-R43, and the region previously identified in mutational studies linked to antibody binding T50-N56. Within DII we have included the second known epitope C65-E72, the region of high correlation G69-T79, and the second stabilising loop T106-G109 which interacts with K33-Y36. Finally within DV we have included the final disulfide between C288-C326, the region of high correlation F280-Y290 and the region linked with phospholipid binding K305-A320.


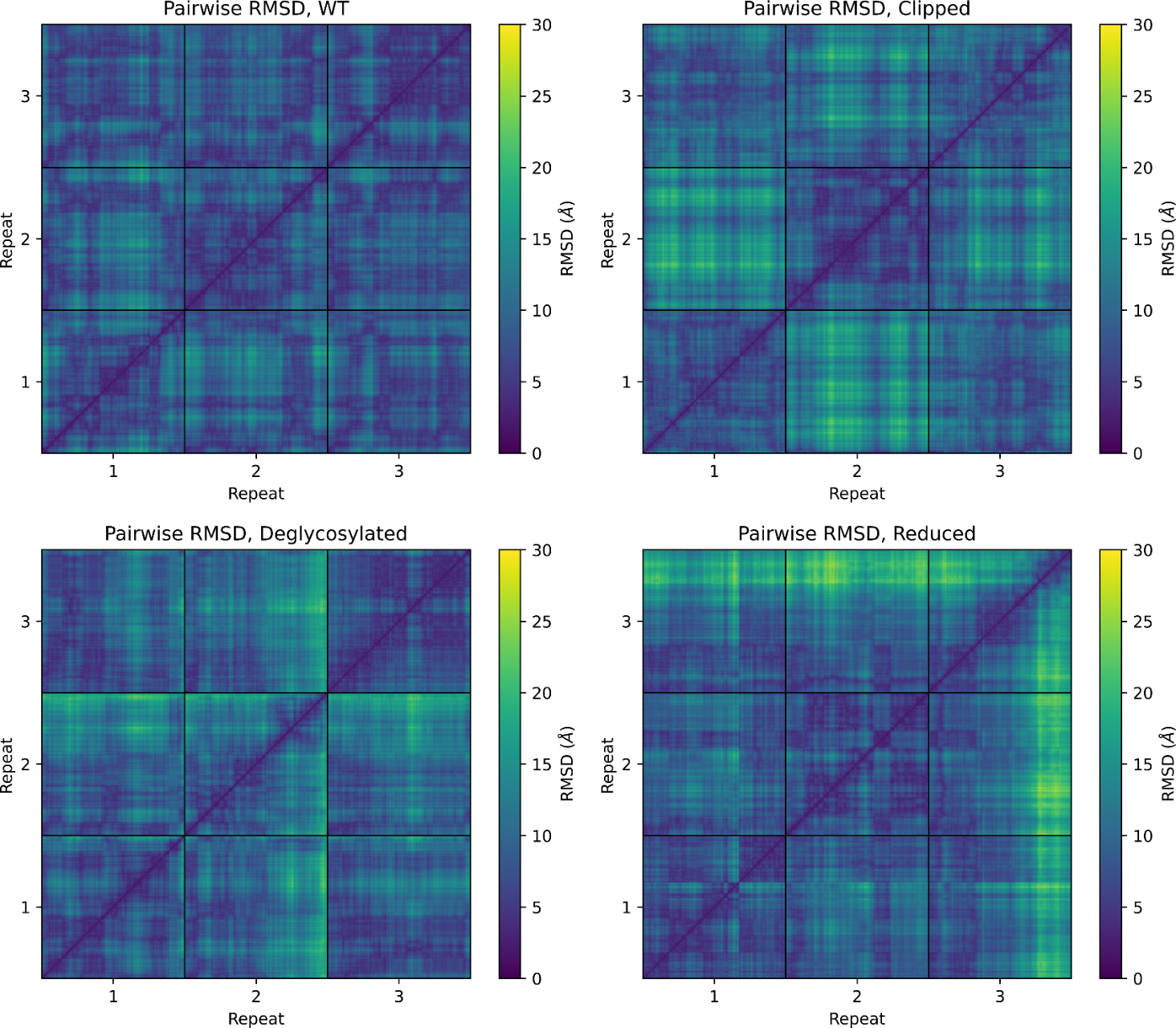


Supp. Figure 2: Pairwise RMSD analysis of every frame against every frame for all four models. This confirms that the simulations have reached a stable state by the end of the 100 ns simulated and that the most stable structures were the wild type and plasmin clipped models; with the highest RMSD values seen, both within and between repeats, for the deglycosylated and reduced simulations.


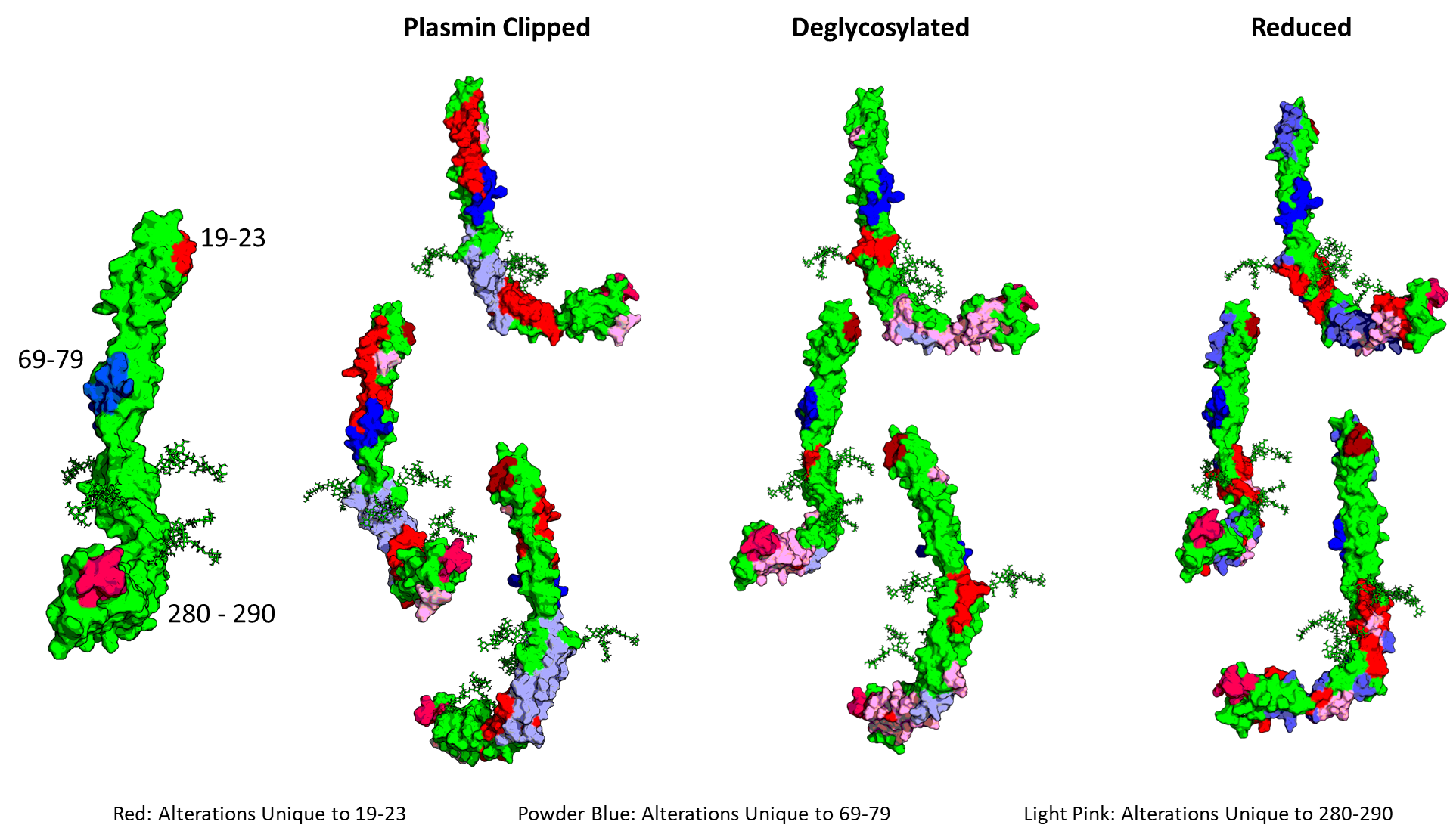


Supp. Figure 3: Showing the different sites for changes in correlation across the protein. As can be seen far more alterations in regulation are seen in the 1st 2 domains (DI-DII) suggesting more alterations in the epitope region. Similarly in the reduced protein some changes are seen in the 1st domain but these are driven by the 69–79 region rather than the 19–23 region as in plasmin clipped. The deglycosylated shows regulation in the 4th and 5th domains mainly driven by the 280–290 region.


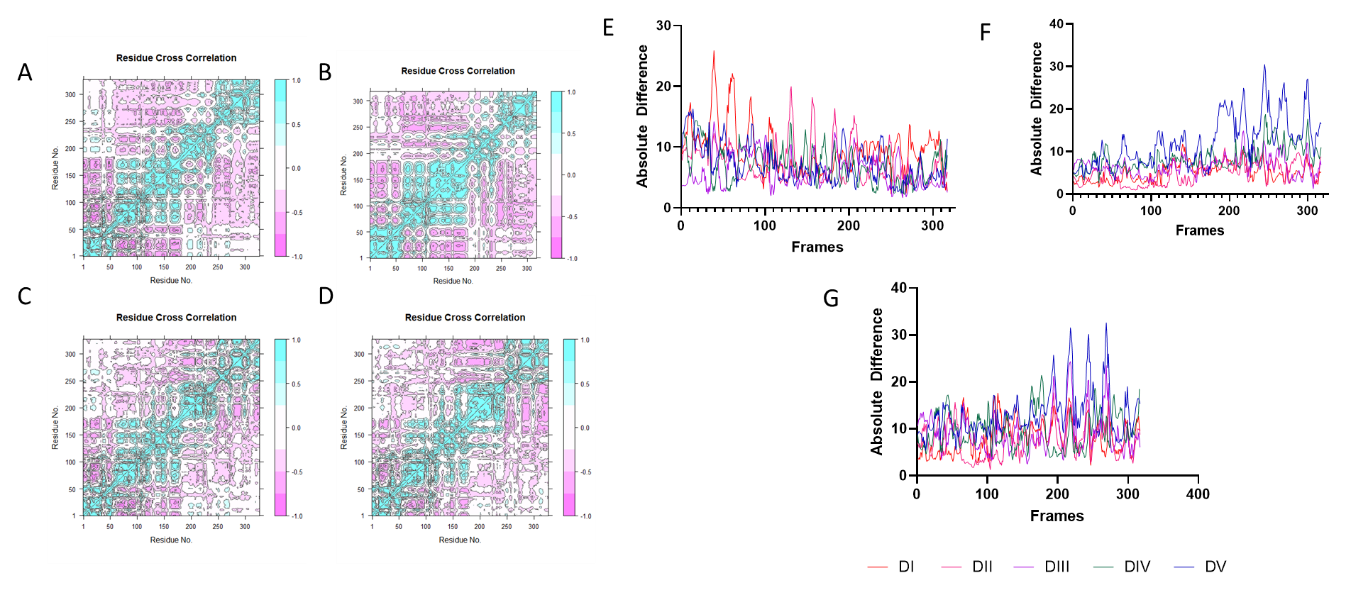


Supp. Figure 4: DCCM Analysis of the simulations. Panel A shows the DCCM analysis of β2GPI with wild type species, whilst B shows the equivalent for Plasmin Clipped β2GPI and C for deglycosylated β2GPI and D for reduced β2GPI. As can be seen there is a strong positive correlation diagonally in A with a strong negative correlation within domains I to III, which then weakens for DIV and V. Further to this, some long-range association is seen from DIV to DI. Panel E shows the absolute difference between wild type and Plasmin Whilst F shows the cumulative difference in wild type vs Deglycosylated and G shows the cumulative difference in reduced protein. Interestingly, despite the cleavage of the terminal 8 amino acids in the 5th domain, the biggest difference in correlation is seen with DI, specifically DI internally and vs the DI-DII region. In contrast F shows deglycosylated differences and the biggest differences are seen in DV when correlating with DV and DIV, this is interesting given the modifications for this variant take place in the 3rd and 4th domain. Finally, the reduced (G) shows differences in all correlations for DV and DIV between P200-K268 correlating with all the domains, this is interesting as the modification for this is in the terminal 8 amino acids too.


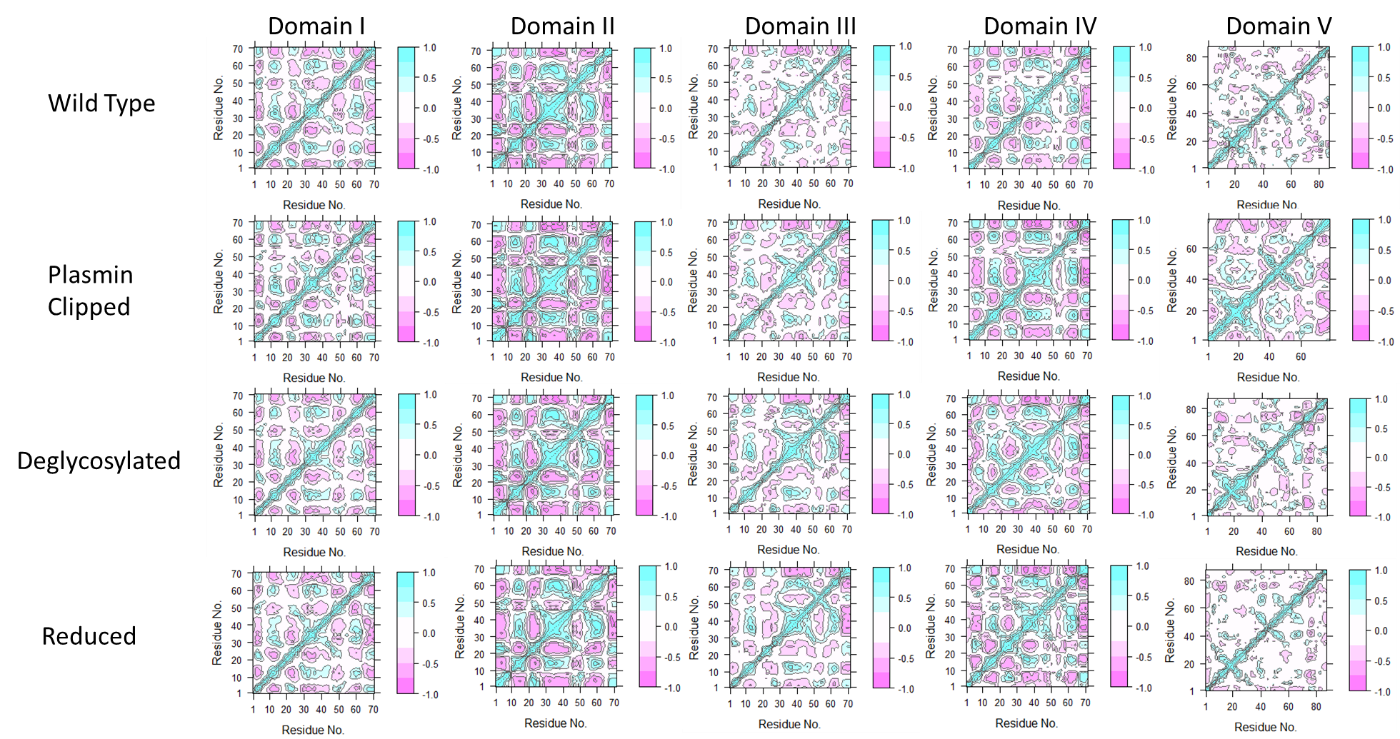


Supp. Figure 5: We then conducted individual DCCM analysis to see if there were dysregulations within domains for each of the proteins. As can be seen, correlations are greatly increased in DV when plasmin cleavage has taken place, similarly, DIV becomes more strongly regulated in all the variants in comparison to wild type. In all the variants DII is the most internally regulated with correlations either negatively or positively being at extremes for most amino acids, this contrasts with the largely dysregulated DV. It is interesting to see in plasmin clipped DI also becomes more tightly internally regulated, showing there is some link between DI and DV in this co-regulation which extends from the long-range analysis to internal dynamics.


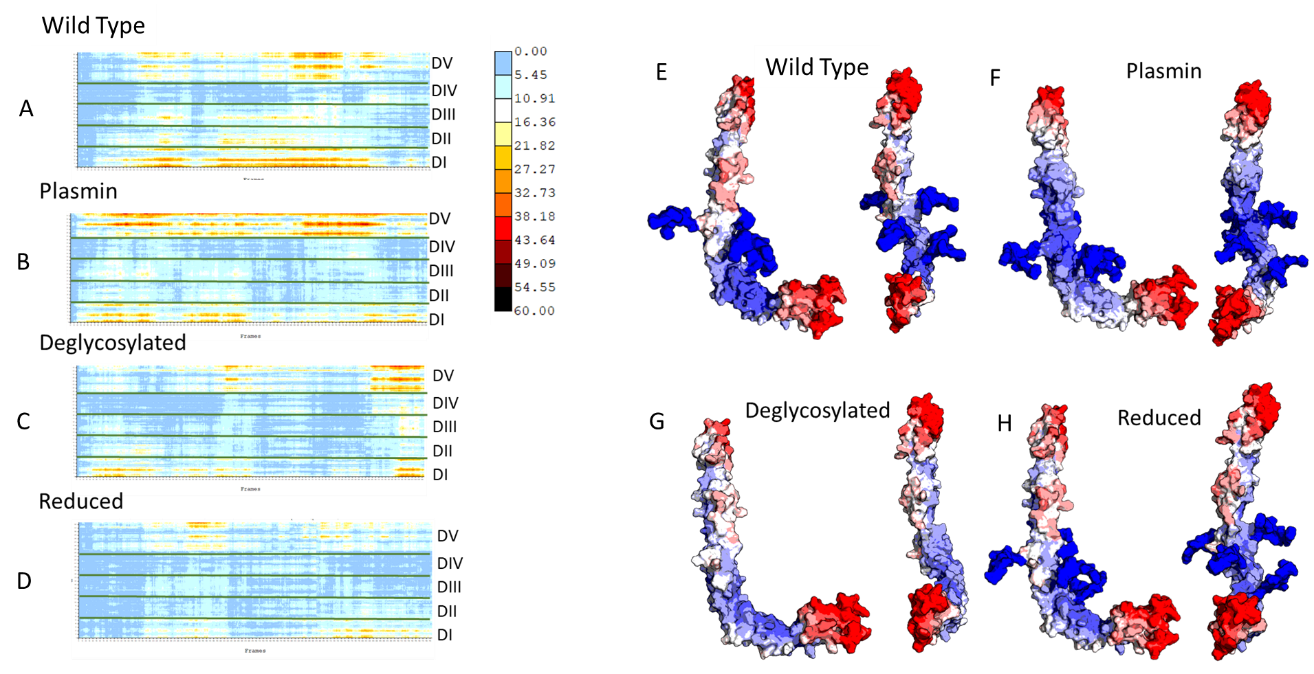


Supp. Figure 6: Plots of the RMSD per amino acid, on the left A-D show the movement maximised to 60, as can be seen increased movement is observed for wild type, deglycosylated and reduced in the 1st and 5th domains, whilst in plasmin clipped the most extreme movement is seen in DV. These sites have been mapped onto models E-H, the colouring has been defined with a maximum of 2x the Standard deviation of the mean RMSD and a minimum of 2x the maximum of the standard deviation for each protein. As can be seen the movement is dominated by the terminal domains for all species.


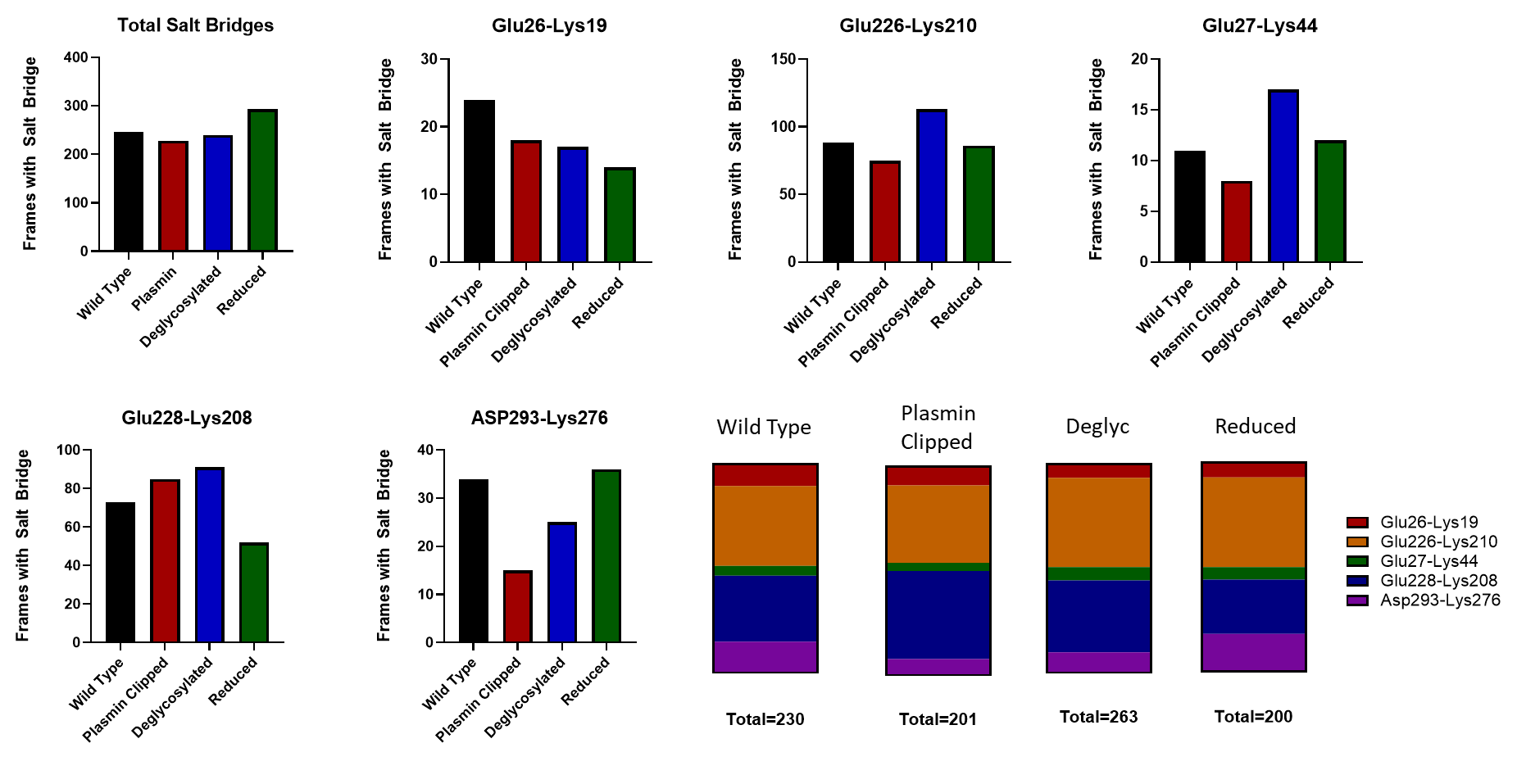


Supp. Figure 7: Analysis of salt bridges across the simulations showed increased salt bridges in E26-K19 for wild type, whilst deglycosylated showed increased formation at E27-K44. The proportion of salt bridges also appeared largely similar, however, for plasmin clipped the E228-K208 formed the majority of its bridges in contrast to wild type which showed E226-K210 as its dominant bridge. The loss of the bridge in D293-K276 may explain the increased movement seen in the plasmin clipped form in the 5th domain.


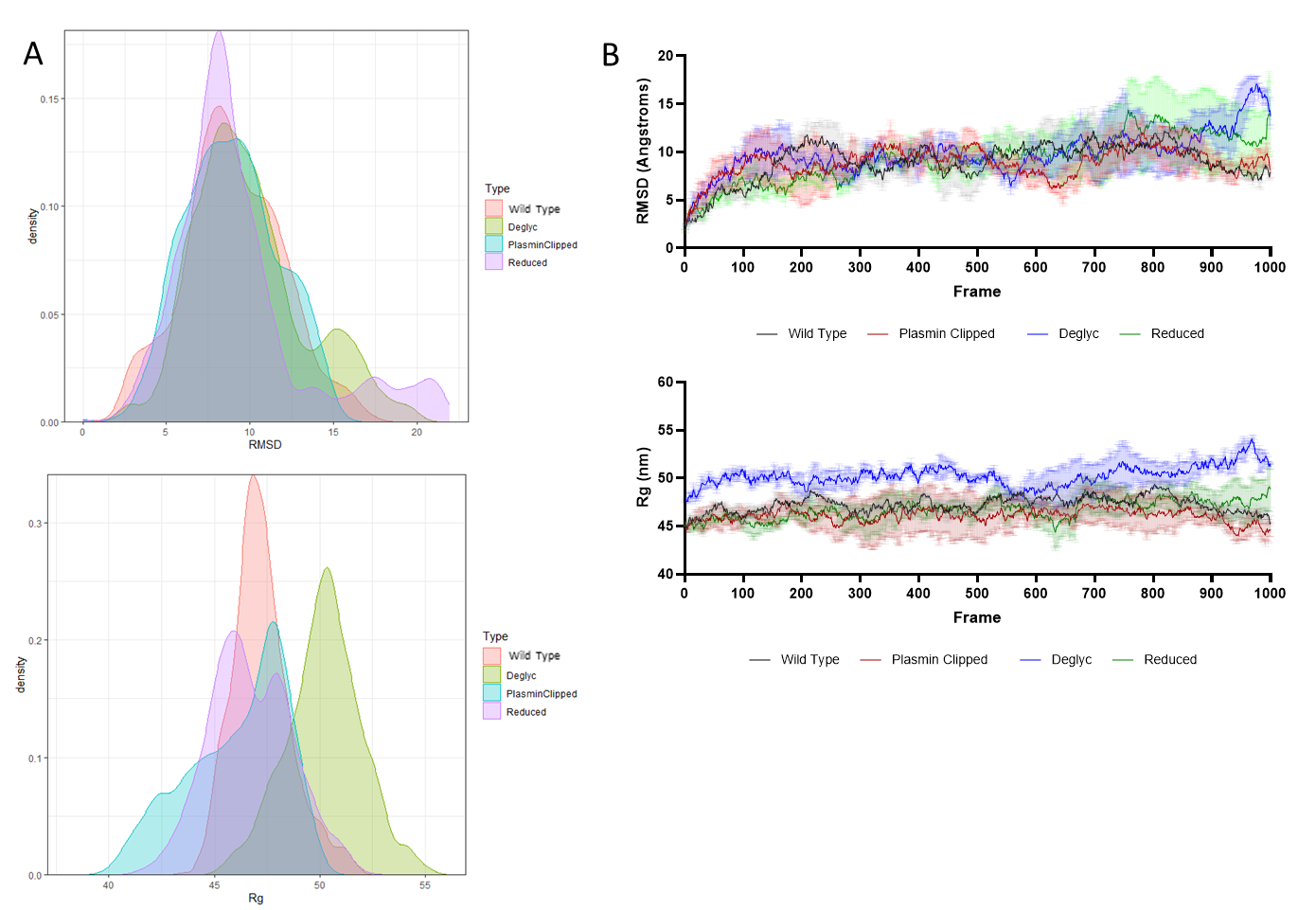


Supp. Figure 8: Assessment of overall movement showed modification of β2GPI lead to altered RMSD profiles (panel A). Histograms suggested the most extreme forms would be within the reduced and deglycosylated forms, with analysis over time averaged across 3 repeats (panel B) suggesting the most extreme forms are generated after 700 frames. Analysis of the radius of gyration suggested the smallest forms to be within the plasmin clipped species and the largest within the deglycosylated. This suggests the glycans play a role in stabilising the J shape, whilst analysis over time showed the plasmin clipped species becomes smaller early (~ 250 frames) and remains that way in contrast to the deglycosylated which is larger throughout.
